# Molecular diagnostics in cancer patients with suspected respiratory mold infections

**DOI:** 10.1128/jcm.01201-25

**Published:** 2025-11-26

**Authors:** V. Rickerts, J. Springer, J. Gerkrath, D. Korczynski, J. Kessel, I. Wieters, T. Liebregts, J. Steinmann, O. A. Cornely, P. Koehler, S. Schwartz, T. Elgeti, L. Meintker, S. W. Krause, J. Held, W. J. Heinz, B. Willinger, G. Maschmeyer, S. Voigt, J. Reiche, J. J. Vehreschild, H. Einsele, D. Teschner, J. Löffler

**Affiliations:** 1 Robert Koch-Institut, Unit 16: Mycotic and Parasitic Agents and Mycobacteria, Berlin, Germany; 2 Medizinische Klinik und Poliklinik II, Wü4i, Würzburg University Hospital 368109 , Würzburg, Bayern, Germany; 3 Department of Hematology, Medical Oncology, and Pneumology, University Medical Center of the Johannes Gutenberg University 39068, Mainz, Germany; 4 Medical Clinic 2, Goethe University Frankfurt, Frankfurt, Hessen, Germany; 5 Robert Koch-Institut, Unit 37: Healthcare-Associated Infections, Surveillance of Antibiotic Resistance and Consumption 9222, Berlin, Germany; 6 Department of Hematology and Stem Cell Transplantation, University Hospital Essen 39081 , Essen, Nordrhein Westfalen, Germany; 7 Institute of Medical Microbiology, Excellence Center for Medical Mycology (ECMM), University Hospital Essen 39081 , Essen, Nordrhein Westfalen, Germany; 8 Institute of Clinical Microbiology, Infectious Diseases and Infection Control, Paracelsus Medical University, Klinikum Nürnberg 470426 , Nürnberg, Germany; 9 Institute of Translational Research, Cologne Excellence Cluster on Cellular Stress Responses in Aging-Associated Diseases (CECAD), University of Cologne 14309, Cologne, Nordrhein Westfalen, Germany; 10 Department I of Internal Medicine, Center for Integrated Oncology Aachen Bonn Cologne Duesseldorf (CIO ABCD), University of Cologne 14309, Cologne, Nordrhein Westfalen, Germany; 11 Department I of Internal Medicine, Excellence Center for Medical Mycology (ECMM), University of Cologne 14309, Cologne, Nordrhein Westfalen, Germany; 12 Partner Site Bonn-Cologne, German Centre for Infection Research, Cologne, Nordrhein Westfalen, Germany; 13 Clinical Trials Centre Cologne (ZKS), University Hospital Cologne, University of Cologne 27182, Cologne, Nordrhein Westfalen, Germany; 14 Department I of Internal Medicine, Center for Integrated Oncology Aachen Bonn Cologne Duesseldorf (CIO ABCD), University of Cologne 14309, Cologne, Germany; 15 Department I of Internal Medicine, Division of Clinical Immunology, University of Cologne 14309, Cologne, Nordrhein Westfalen, Germany; 16 Department of Hematology, Oncology and Cancer Immunology (Campus Benjamin Franklin), Universitätsmedizin Berlin, corporate member of Freie Universität and Humboldt-Universität zu Berlin, Berlin, Berlin, Germany; 17 Klinik für Radiologie, Charité—Universitätsmedizin Berlin, corporate member of Freie Universität and Humboldt-Universität zu Berlin, Berlin, Berlin, Germany; 18 Abteilung für Innere Medizin 5, Uniklinikum Erlangen, Erlangen, Bayern, Germany; 19 Mikrobiologisches Institut—Klinische Mikrobiologie Immunologie und Hygiene, Universitätsklinik Erlangen und Friedrich-Alexander-Universität Erlangen-Nürnberg, Erlangen, Bayern, Germany; 20 Medizinische Klinik 2, Caritas Hospital 39646, Bad Mergentheim, Bayern, Germany; 21 Division of Clinical Microbiology, Department for Laboratory Medicine, Medical University of Vienna 27271, Vienna, Austria; 22 Department of Hematology, Oncology and Palliative Care, Klinikum Ernst von Bergmann 14959, Potsdam, Brandenburg, Germany; 23 Department of Hematology, Oncology and Tumor Immunology, Charité University Medicine Berlin, Berlin, Germany; 24 Robert Koch-Institut, Unit 12: Measles, Mumps, Rubella and Viruses Affecting Immunocompromised Patients, Berlin, Germany; 25 Robert Koch-Institut, Unit 17: Influenza and Other Respiratory Viruses, Berlin, Germany; University of Utah, Salt Lake City, Utah, USA

**Keywords:** *Aspergillosis*, mucormycosis, PCR, FISH, BALF, cancer, CMV

## Abstract

Invasive fungal infections (IFIs), including aspergillosis and mucormycosis, are serious complications in cancer patients. Reliable detection of etiologic agents is challenging but important for optimal patient management. In a prospective multicenter study, we compared fungal detection by culture, specific and unspecific qPCRs, and fluorescence *in situ* hybridization (FISH) targeting *Aspergillus* and Mucorales in bronchoalveolar lavage fluid (BALF) and serum of adult cancer patients with suspected fungal pneumonia. In subgroups of patients, fungal DNA was amplified from serum. We evaluated 210 IFI episodes (proven: 3, probable: 72, possible: 107, unclassifiable: 28). Broad-range fungal PCR from BALF was terminated early due to frequent amplification of colonizing yeasts. Specific qPCR assays targeting molds (*Aspergillus,* Mucorales) were superior for mold detection (26%) compared to FISH (15%) and culture (8%). Detection of fungi by FISH predicted subsequent fungal culture positivity. In proven/probable IFI episodes, detection of *Aspergillus* DNA was more likely in BALF than in serum (35/75 [47%] vs 11/66 [17%]; *P* = 0.0002) in contrast to Mucorales DNA (7/75 [9%] vs 5/66 [8%]; *P* = 0.73). Mucorales DNA was detected together with *Aspergillus* DNA in 6 of 7 BALF samples but not in serum. Specific qPCRs targeting *Aspergillus* and Mucorales from BALF are the most sensitive means to identify molds in adult cancer patients with suspected fungal pneumonia. BALF qPCRs suggest frequent fungal coinfection, while viruses were rarely detected. Fungal detection by FISH may predict subsequent positive fungal cultures.

**IMPORTANCE:**

Mold pneumonia is a serious complication in cancer patients. Reliable detection of etiologic agents is critical for patient care, but optimal testing strategies are undefined. We performed a prospective study comparing molecular tests (specific and unspecific qPCR and FISH) and culture on bronchoalveolar lavage fluid (BALF) and serum, focusing on aspergillosis and mucormycosis to gain insights into mold detection in high-risk patients. We demonstrate that FISH visualizes fungal elements in BALF, predominantly conidia, suggesting recent fungal exposure or failure to clear inhaled conidia at the time of diagnostic testing. We find that specific qPCRs from BALF are the most sensitive method to detect *Aspergillus* and Mucorales, superior to broad-range qPCR, culture, and testing of serum. Importantly, Mucorales DNA is mostly co-detected with *Aspergillus*, suggesting co-infections. Although DNA detection in serum is more likely in mucormycosis than in aspergillosis, sole serum testing may miss mucormycosis, precluding optimal patient care.

## INTRODUCTION

Pneumonia caused by molds is a serious complication in patients with severe underlying diseases including hematologic malignancies and the critically ill (1). In these patients, aspergillosis is the most prevalent invasive mold infection diagnosed (2). Other mold infections, including mucormycosis, may not be differentiated from aspergillosis by clinical presentation but require unique management strategies due to antifungal resistance and rapid disease progression. In addition, viral infections are increasingly recognized as risk factors for respiratory mold infections and may be a target for specific intervention to improve patient outcome (3).

While *Aspergillus* detection by molecular tests or antigen detection identifies these fungi with a higher sensitivity as compared to culture and microscopy in bronchoalveolar lavage fluid (BALF), optimal diagnostic strategies to identify a broader spectrum of molds remain undefined. Besides mycological culture, specific qPCR assays, broad-range qPCR with amplicon sequencing, and fluorescence *in situ* hybridization (FISH) targeting ribosomal RNA (rRNA) represent sensitive molecular detection methods. As FISH targeting rRNA does not require previous amplification of the molecular target due to a high number of ribosomes in replicating fungi, it has the potential to be a sensitive, specific, and rapid diagnostic tool, but it has not been studied for the diagnosis of mold infections using BALF.

We compared different molecular strategies, that is, broad-range fungal qPCR with amplicon sequencing, specific real-time qPCR assays targeting *Aspergillus* and Mucorales, and FISH detection of *Aspergillus* and Mucorales with fungal cultivation using diverse media from BALF to identify the etiology of invasive pulmonary mold infections in cancer patients to improve patient care. In subgroups of patients, fungal DNA was amplified by qPCR from serum to compare results with BALF testing.

## MATERIALS AND METHODS

### Study population and ethics approval

We conducted a prospective multicenter study at 10 clinical sites and 2 central diagnostic laboratories performing qPCR (Wü4i, University of Würzburg), mycological culture, fungal FISH, and viral diagnostics (Robert Koch-Institut). The study was approved by the ethics committee of the University Hospital Würzburg (#270/15).

After informed consent, adult cancer patients with new or worsening pulmonary infiltrates unresponsive to antibacterial therapy, suspicious for an invasive mold infection, were included. Clinical data (underlying disease, risk factors for mold infections, radiologic findings, antimicrobial therapy) were documented at participating centers in a web-based database at the time of inclusion and around day 90 (ClinicalSurveys.net).

BALF samples were collected according to local standards and sent to both central laboratories for diagnostic testing in addition to local center-specific diagnostic assessment procedures.

**Fig 1:**
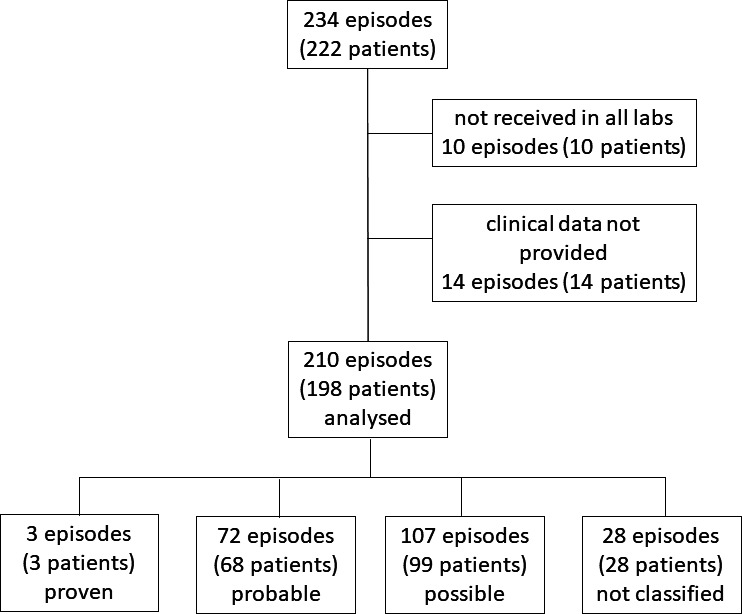
Classification of enrolled patients and respective episodes of suspected respiratory mold infections according to EORTC/MSG 2008 criteria.

In total, 234 episodes in 222 patients were included between May 2016 and May 2018. More than one episode per patient was allowed after resolution of clinical signs from the previous episode at the investigator’s discretion. Ten episodes from 10 patients were excluded from the analysis as BALF was not received in both central laboratories, and 14 samples from 14 patients were excluded because baseline clinical data were not reported (Fig. 1). In a subgroup of patients (*n* = 175), serum was collected between 3 days each prior and after BALF sampling, stored frozen, and later analyzed by fungal qPCR.

Fungal diseases were classified according to the EORTC/MSG criteria as published in 2008 (4). Microbiologic criteria included cultivation of a mold pathogen, microscopy demonstrating mold hyphae or positive galactomannan from BALF.

### Laboratory procedures

For cultivation of fungi, BALF was centrifuged (1,600 *g* for 10 min), and cultivation of the pellet was performed on Sabouraud-Dextrose agar, *Candida* ID agar, and inhibitory mold agar (IMA) (all from Becton-Dickinson, Heidelberg, Germany); Birdseed agar for identification of melanin-producing *Cryptococcus*; *Scedosporium* select agar containing benomyl; and Sabouraud-Dextrose broth (all manufactured at RKI), using 50 µL for each medium (300 µL total volume used for cultivation), and cultures were incubated at 37°C and 26°C (Birdseed agar only). Plates were checked daily for growth for 14 days. Yeast isolates were identified by matrix-assisted laser desorption/ionization time-of-flight and molds by phenotype and sequencing of the ITS region, except for *Aspergillus*, which was identified by sequencing of a part of the beta-tubulin gene. Cultivated *Aspergillus fumigatus* was genotyped by cell surface protein typing (5).

DNA extraction for fungal PCR testing was performed by combining BALF supernatant and pellet fraction using bead-beating and a commercial kit as described previously (6). Briefly, both BALF fractions were separated after centrifugation. The pellet fraction only was bead-beaten to free cell-associated DNA into the liquid phase. Both fractions were re-united, and cell-free DNA was extracted using a commercial kit. DNA from serum was extracted as described before (7). Specific qPCR assays were used to detect DNA of *Aspergillus* with melt curve analysis for species identification (8, 9) and Mucorales with species identification using Sanger sequencing (10, 11). A broad-range assay targeting a region of the 28S rRNA gene using primers 12f and 13r was performed in the initial samples (*n* = 42) but stopped due to a low diagnostic yield (12). Human DNA was quantified as described before by targeting the human ribosomal RNA gene (6, 13). DNA extraction from serum and qPCR specific for *Aspergillus* were compliant with the recommendation of the fungal PCR initiative (FPCRI) (14). Appropriate controls were included to document efficient DNA extraction, absence of PCR inhibition, and contaminating fungal DNA (7). Amplicons were identified by Sanger sequencing (Mucorales and broad-range PCR) (10).

GM was quantified from BALF using ELISA (Platelia Antigen ELISA, BioRad, Feldkirchen, Germany) according to manufacturer’s instructions. Positive samples were defined by optical density index of ≥0.5 (BALF) and ≥0.5 (serum).

FISH was performed from BALF pellets after incubation of 1 mL with 2 mL of paraformaldehyde (PFA) for 30 min at room temperature. Fixed BALF (300 µL) was spotted on two microscopy slides using a cytospin centrifuge. The first slide was incubated with hybridization buffer containing a specific probe targeting *Aspergillus* with the Cy3 dye, two probes targeting common agents of mucormycosis with the Cy5 dye, and the unspecific probe EUK 516 targeting eukaryotic cells with the dye Alexa Fluor 488 (15, 16). The second slide was incubated with the non-sense probe EUB coupled with Cy3, Cy5, and Alexa Fluor 488 to control for autofluorescence and unspecific hybridization as a negative control. Hybridization, post-hybridization washes, and slight preparation for microscopy were performed as reported previously (16). Epifluorescence microscopy was performed as described previously (17). Images were taken by a technician blinded for clinical data, culture, and PCR results. Positive hybridization was assumed when fungal elements showed a probe signal in the absence of evidence for unspecific hybridization or autofluorescence in the negative controls with non-sense probes. Control fungal elements (*Candida parapsilosis* yeast cells, *A. fumigatus* conidia and hyphae, conidia and hyphae of *Lichtheimia corymbifera*) grown overnight in liquid broth and fixed with PFA were included in each experiment as positive control and documentation of specific hybridization.

Additional methods including testing for viruses, *in vitro* susceptibility testing, and typing of *A. fumigatus* are presented in the Supplemental file.

### Statistical analysis

Statistical analyses were performed in GraphPad Prism 9.1.0 for Windows (GraphPad Software, San Diego, USA) and Stata 17 (Stata Corp., College Station, Texas, USA).

## RESULTS

### Patient characteristics

The final analysis included 210 pneumonia episodes in 198 patients (Fig. 1). Two patients provided three samples, eight patients provided two samples from subsequent pneumonia episodes.

Patients’ underlying diseases were mostly hematological malignancies, predominantly acute leukemias with chemotherapy-associated neutropenia or stem cell transplantation (Table 1). In 13 episodes, medical history included a mold infection (aspergillosis: 12, mucormycosis: 1) that had resolved before inclusion into the study. A probable/proven fungal infection was subsequently diagnosed in 8/13 (62%) vs 67/197 (34%) (*P* = 0.05). Antibacterial agents during the 72 h before bronchoscopy were administered in 79%, antivirals in 31%, and non-mold active antifungals in 6% of all episodes. Mold active antifungals were administered in 58% of patients for a median of 18 (range: 1–351) days (Table 1).

**TABLE 1:** Clinical characteristics of included patients and the subgroup with proven or probable mold infection^*a*^

	All, *n* = 210 (%)	Proven/probable, *n* = 75 (%)
Sex		
Female	77 (37)	26 (35)
Male	133 (63)	49 (65)
Age, years (median, min–max)	57 (19–84)	57 (24–82)
Underlying condition		
AML	95 (45)	31 (41)
ALL/NHL/CLL	56 (27)	17 (23)
Multiple myeloma	13 (6)	5 (7)
Other hematologic malignancy	40 (19)	18 (24)
Other malignancy	6 (3)	4 (5)
Risk factors for fungal infection		
Neutropenia	146 (70)	50 (67)
Duration (days)	30 (1–365)	30 (2–180)
Immunosuppressive therapy	93 (44)	33 (44)
Stem cell transplantation	80 (38)	29 (39)
Autologous	12 (6)	2 (3)
Allogeneic	68 (32)	27 (36)
GvHD	33 (16)	11 (15)
Previous mold infection		
Aspergillosis	12 (6)	7 (9)
Mucormycosis	1 (0.5)	1 (1)
Radiologic findings		
Halo	59 (28)	27 (36)
Inverse halo	6 (3)	2 (3)
Pleural effusion	87 (41)	37 (49)
Abnormal sinus CT	7 (3)	4 (5)
Anti-infectives at day of bronchoscopy		
Antibacterial	165 (79)	62 (83)
Antifungal, mold-active^*b*^	121 (58)	51 (69)
Antifungal, not mold-active^*c*^	13 (6)	4 (5)
Antiviral	68 (32)	19 (25)

^*a*^ ALL, acute lymphogenous leukemia; NHL, non-Hodgkin’s lymphoma; CLL, chronic lymphocytic leukemia; GvHD, graft vs host disease; CT, computed tomography.

^*b*^ Voriconazole, posaconazole, isavuconazole, caspofungin, anidulafungin, micafungin, liposomal amphotericin B.

^*c*^ Fluconazole.

### Mycologic testing from BALF

**Fig 2:**
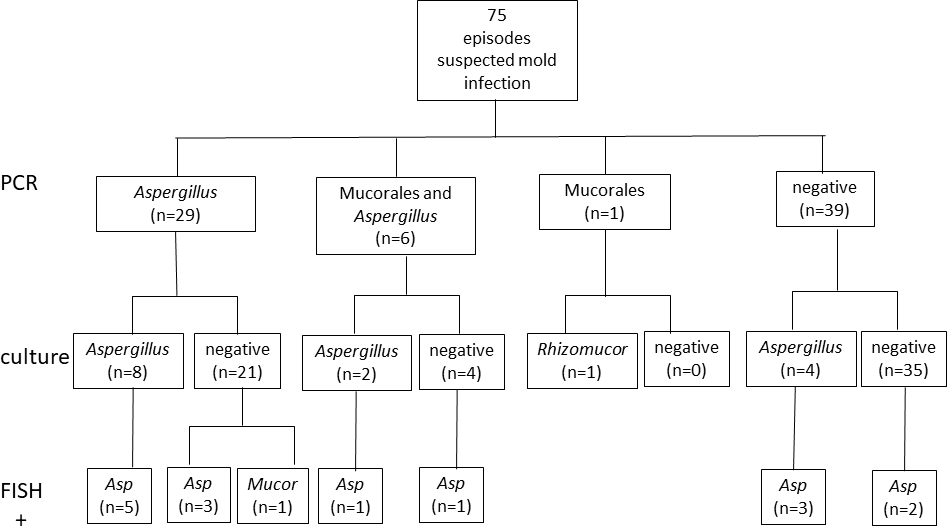
Comparison of PCR, culture, and FISH positivity for detection of *Aspergillus* and Mucorales in BALF samples from proven (*n* = 3) or probable (*n* = 72) episodes. Abbreviations: FISH, fluorescence *in situ* hybridization; Asp, *Aspergillus*; Mucor, Mucorales; +, positive.

Microbiologic laboratory results are summarized in Table 2 for all samples (*n* = 210) and for the subgroup from proven/probable invasive fungal infection (IFI) episodes (*n* = 75) (Fig. 2). For a subgroup of episodes (*n* = 187), *Aspergillus* GM antigen was tested from BALF (Table 2).

**TABLE 2:** Microbiologic results of BALF samples from all episodes proven or probable episodes and serum marker performed in a subgroup of patients

	All episodes, *n* = 210 (%)	Probable/proven, *n* = 75 (%)
Fungal culture		
Yeasts	67 (32)	30 (40)
Molds	23 (11)^*a*^	17 (23)^*a*^
*A. fumigatus*	15 (7)	14 (19)
*Rhizomucor*	1 (0.5)	1 (1)
Other^*b*^	7 (3)	2 (3)
Specific qPCR		
*Aspergillus*	45 (21)	35 (47)
*A. fumigatus*	38 (18)	29 (39)
*A. terreus*	2 (1)	2 (3)
*A. flavus*	1 (0.5)	1 (1)
Unspecified	4 (2)	3 (3)
Mucorales	10 (5)^*c*^	7 (9)^*c*^
*Rhizopus*	5 (2)	4 (5)
*Rhizomucor*	3 (1.5)	3 (4)
*Mucor*	2 (1)	0 (0)
Fungal FISH		
*Aspergillus* probe	26 (12)	15 (20)
Mucorales probe	6 (3)	1 (1)
Yeast^*d*^	71 (34)	32 (43)
*Aspergillus* GM antigen positive (>0.5)	36/187 (19)	33/66 (50)
Virus PCR		
Respiratory	33/191 (17)	8/70 (11)
CMV	26/166 (16)	9/57 (16)
Serum marker		
*Aspergillus* PCR	15/175 (9)	11/66 (17)
Mucorales PCR	8/175 (5)	5/66 (8)
Both positive	0/175 (0)	0/66 (0)

^*a*^ In 10 of 23 (43%) and 8 of 17 (44%) samples with the growth of molds, additional yeasts were cultivated.

^*b*^ Other molds included *Talaromyces* sp. (n = 2), *Hypholoma* (n = 2), *Fusarium* (n = 1), and *Aureobasidium* (n = 1).

^*c*^ In 6 of 10 (60%) and 6 of 7 (86%) cases, Mucorales DNA was amplified together with *Aspergillus* DNA.

^*d*^ Unspecific probe and yeast morphology.

Culture was less likely to demonstrate growth of *Aspergillus* than specific *Aspergillus* qPCR from BALF in all episodes (15 [7%] vs 45 [21%]; *P* = 0.0001) and in probable/proven episodes (14 [19%] vs 35 [47%]; *P* = 0.07). BALF with cultivation of *Aspergillus* demonstrated lower sample quantification cycles (Cqs) in qPCR (*n* = 14; mean = 40.9; 95% CI = 35.6–46.2) than in culture-negative (*n* = 35; mean = 43.6; 95% CI = 41.7–45.6) samples. *A. fumigatus* was cultivated on IMA in 9/15 (60%) cases and Sabouraud-Dextrose agar in 7/15 (47%) culture-positive samples. Sequencing of cultivated *Aspergillus* confirmed *A. fumigatus sensu stricto* in all cases.

Culture was less likely to demonstrate growth of Mucorales than specific qPCR from BALF in all episodes (1 [0.5%] vs 10 [5%]; *P* = 0.048) and in probable/proven episodes (1 [1%] vs 7 [9%]; *P* = 0.09). Mucorales DNA was detected at Cq 29.95 in a case with cultivation of *Rhizomucor pusillus* on IMA only. Culture-negative samples demonstrated different amounts of DNA (*n* = 9; mean = 36.49; 95% CI = 30.25–42.73). Of note, Mucorales DNA was detected in 6 of 7 BALF (86%) of patients with probable/proven infection together with *Aspergillus* DNA suggesting coinfection.

Broad-range fungal PCR was performed in a subgroup of BALF samples (*n* = 42) and was positive in 37 (88%). In 10 (27%) of these samples, specific qPCRs identified DNA of a mold pathogen (*Aspergillus* 9; Mucorales 2). In only one of these samples, sequencing of the broad-range amplicon identified *Aspergillus* as the specific qPCR detected. Here, *Aspergillus* was also cultivated from BAL, and *Aspergillus* DNA was also amplified from blood. In contrast, sequencing identified yeasts that were also cultivated from BAL in six samples. In three samples, the amplicon of the broad-range qPCR could not be identified due to mixed sequence reads. This suggests a high rate of detection of colonizing yeasts by broad-range qPCR rather than amplification of mold DNA. The frequent occurrence of yeasts in BALF was confirmed by culture (67/210; 32%) and FISH (71/210; 34%).

Inhibition of the PCR reaction was detected in 5 of 210 (2%) BALF samples and in 5 of 117 (4.3%) serum samples. One *Aspergillus* qPCR in BALF was positive in the context of detected PCR inhibition. No sample showed growth of a mold and negative mold qPCR in the context of PCR inhibition.

The mean human DNA amount in BALF was 7.1 ng/mL (140 ng/mL to 1,043 µg/mL). The median human DNA content was higher in non-neutropenic episodes (*n* = 64) than in neutropenic episodes (*n* = 146) (13 µg/mL vs 5.2 µg/mL BALF; *P* = 0.001). For patients with neutropenia, a human DNA content >14 µg/mL BALF was associated with a higher likelihood of positive *Aspergillus* qPCR (30% vs 15%; *P* = 0.09).

**Fig 3:**
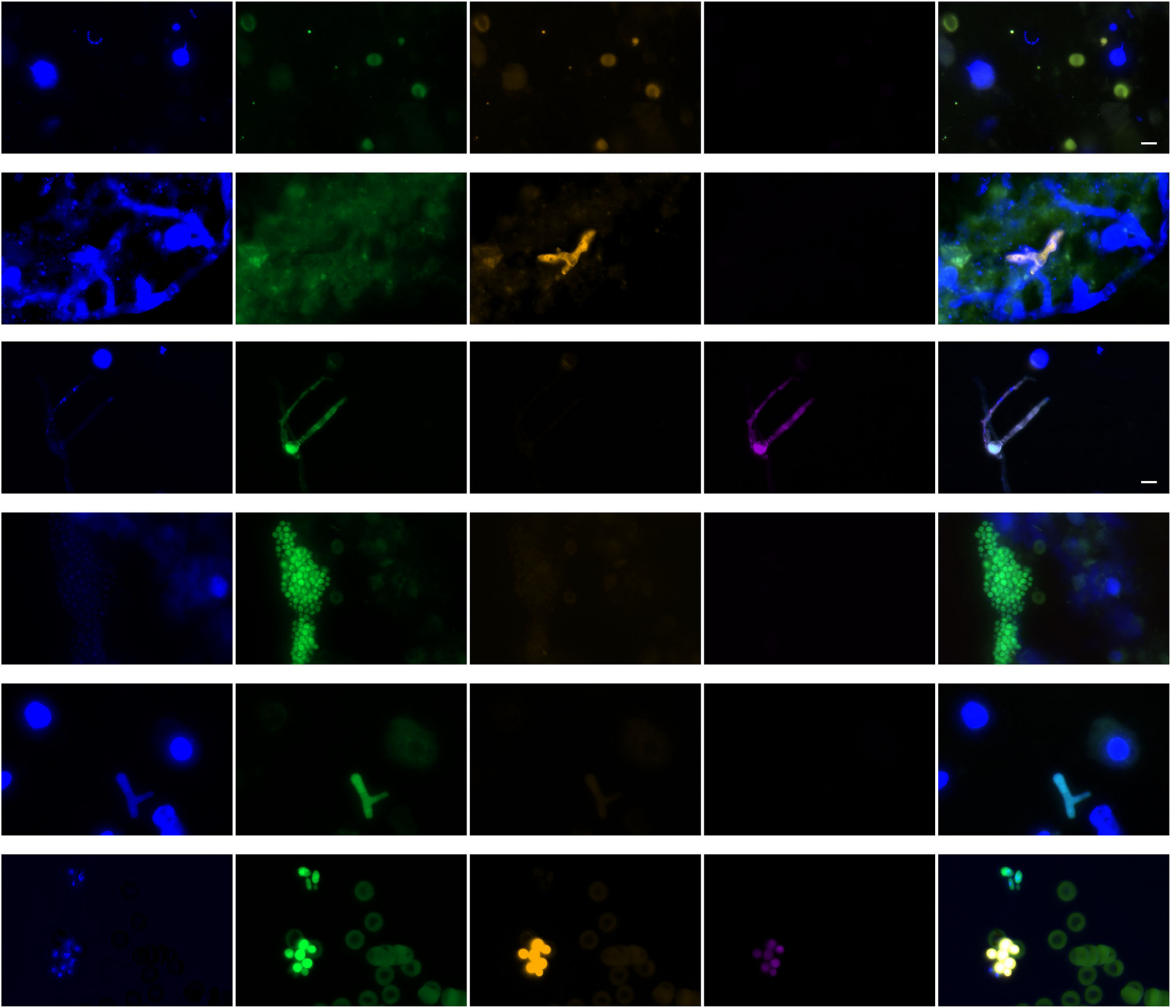
Fluorescence microscopy of BALF samples (each row represents one sample) using DAPI staining (targeting DNA) and FISH targeting ribosomal RNA by an unspecific probe (EUK) and specific probes targeting *Aspergillus* using the Cy3 dye and Mucorales using the Cy5 dye**.** First row: conidia, hybridizing with *Aspergillus* probe with leuco- and erythrocytes (autofluorescence Cy3 and FITC). Second row: hyphae, hybridizing with *Aspergillus* probe. Third row: germinating conidia hybridizing with Mucorales probe. Fourth row: yeast cells hybridizing with unspecific probe (EUK) only. Fifth row: hyphal fragment without hybridization of *Aspergillus* and Mucorales probes. Sixth row: yeasts hybridizing with unspecific probe and swollen conidia hybridizing with *Aspergillus* probe. Bar represents 5 µm. Abbreviations: DAPI, 4′,6-diamidino-2-phenylindol; EUK, eukaryotic probe; Cy, cyanine; BALF, bronchoalveolar lavage fluid; FISH, fluorescence *in situ* hybridization.

Culture was less likely to grow *Aspergillus* than FISH to identify *Aspergillus* by hybridization of fungal structures with the *Aspergillus* probe (15 [7%] vs 26 [13%]; *P* = 0.0001) in all episodes but not in probable/proven episodes (14 [19%] vs 15 [20%]; n.s.). FISH targeting *Aspergillus* detected conidia (*n* = 21), hyphae (*n* = 1), or both (*n* = 4) by hybridization (Fig. 3). Culture was less likely to grow Mucorales than FISH to identify Mucorales by hybridization of fungal structures with the Mucorales probe (1 [0.5%] vs 6 [3%]; n.s.) in all episodes and probable/proven episodes (1 [2%] vs 1 [2%]; n.s.). Culture was as likely to grow yeasts as FISH to detect yeast cells by positive hybridization of yeasts with the unspecific probe (67 [32%] vs 71 [34%]; n.s.) in all episodes and in proven/probable episodes (30 [40%] vs 32 [43%]). Detection of *Aspergillus* and *Candida* by FISH was predictors of subsequent cultivation of *Aspergillus* (9/15 [60%] vs 5/55 [9%]; *P* = 0.0001) and Candida (18/32 [56%] vs 12/31 [39%]; *P* = 0.01).

Among *Aspergillus* qPCR-positive samples (*n* = 45), FISH detected *Aspergillus* in 11/45 (24%) samples as compared to 15/165 (7%) *Aspergillus* qPCR-negative BALF (*P* = 0.006). *Aspergillus* qPCR Cq values were not different between *Aspergillus* FISH positive (*n* = 11) and negative samples (*n* = 34) (data not shown). Among Mucorales qPCR-positive samples (*n* = 10), FISH detected conidia with hybridization of the Mucorales probe in only one sample (10%) as compared to 5/200 (2.5%) (n.s.).

Detection of *Aspergillus* by culture, specific qPCR, or FISH was not significantly impaired by the presence of systemic mold-active antifungal prophylaxis (data not shown).

Additional results on BALF testing for respiratory viruses and CMV are presented in the supplemental file.

### Mycologic testing from serum

For a subgroup of episodes (*n* = 175), serum samples were investigated by fungal qPCRs, which detected *Aspergillus* (*n* = 15 [9%]) or Mucorales (*n* = 8 [5%]). Among episodes with available qPCRs from serum and BALF (*n* = 117), *Aspergillus* qPCR in serum was more likely in BALF-positive samples (11/33 [33%] vs 2/84 [2%]; *P* = 0.0001). Mucorales qPCR in serum was more likely in BALF-positive samples (5/8 [63%] vs 3/109 [3%]; *P* = 0.0001). In episodes with BALF and serum positive by *Aspergillus* qPCR (*n* = 11), qPCR from BALF became positive before serum (40.66 ± 5.58 vs 43.25 ± 5.35; *P* = 0.12). In episodes positive by Mucorales qPCR from BALF and serum (*n* = 5), qPCR from BALF became positive 2.4 Cq before serum (data not shown).

In proven/probable IFI episodes, detection of *Aspergillus* DNA was more likely in BALF than in serum (35/75 [47%] vs 11/66 [17%]; *P* = 0.0002) in contrast to Mucorales DNA (7/75 [9%] vs 5/66 [8%]; *P* = 0.73).

### Follow-up after BALF testing

Patients were followed for 0–328 (median 88) days after BALF sampling. At day 90, 120 (66%) patients survived, 28 (13%) patients were lost, and 62 episodes (34%) were reported as lethal. A fatal outcome was more likely among proven/probable mold infection (34/75; 45%), in *Aspergillus* or Mucorales BALF qPCR-positive episodes (23/49; 47%), among serum *Aspergillus* or Mucorales qPCR-positive episodes (14/23; 61%), and in culture of *Aspergillus*-positive BALF (10/16; 63%). In contrast, episodes with FISH-positive BALF were reported as lethal in only 11/33 (33%), with CMV detection by PCR (2/6; 33%) and respiratory virus detection (12/33; 36%).

## DISCUSSION

Respiratory mold infections are serious complications in cancer patients, especially those with treatment-associated neutropenia or after allogenic stem cell transplantation. Identification of molds is a prerequisite for optimal patient management as it allows for specific treatment. In the present study, we were able to demonstrate that specific qPCRs targeting *Aspergillus* and Mucorales on BALF are the most sensitive strategy to identify these pathogens in cancer patients with suspected mold infection. Specific qPCRs are superior to cultivation from BALF and qPCR from blood, which showed lower amounts of fungal DNA detected by specific fungal qPCRs, especially in aspergillosis. Broad-range qPCR from BALF rarely identified mold DNA but frequently detected yeast colonization as confirmed by culture and FISH. Mucorales qPCR detected DNA mostly in BALF that was also positive for *Aspergillus* DNA suggesting coinfections, indicating that a broad diagnostic spectrum including Mucorales may benefit patients with fungal pneumonia by a potential for treatment optimization.

Microbiologic laboratory tests including detection of hyphae or cultivation of mold pathogens are considered gold standards in the diagnosis of respiratory mold infections. Methods for cultivation of molds vary widely. Preanalytic issues including BAL sampling, sample handling before cultivation, cultured volume, and duration of cultivation may impact the yield of fungal cultures. Experts report sensitivities below 30% for cultivation of *Aspergillus* from patients with invasive pulmonary aspergillosis (18, 19). Using additional media, we were not able to improve the culture yield. Selective media allow for more sensitive cultivation of slow-growing molds such as *Scedosporium* due to inhibition of *Aspergillus* by benomyl, which is also not active against frequent agents of mucormycosis (20). While important Mucorales are resistant to benomyl, a selective medium did not improve cultivation in our study despite frequent co-occurrence of Mucorales and *Aspergillus* as detected by PCR (21). In addition, IMA and niger seed agar to identify *Cryptococcus* did not improve cultivation of pathogenic fungi in this study with frequent antifungal use at the time of sampling (21).

The detection of mold hyphae by microscopy is thought to be indicative of invasive mold infection in cancer patients. A rapid differentiation between hyphal morphologies such as septate and non-septate hyphae helps guide patient management and therefore is indicated for many samples including BALF when fungal etiology needs to be considered (22). However, as species resolution by microscopy is limited and small particles such as conidia are difficult to identify in standard fungal staining, we evaluated FISH targeting fungal rRNA to identify fungal elements in BALF. We regularly detected fungal elements, including yeast cells, conidia of mold pathogens, but only rarely mold hyphae. While mold hyphae visualized by FISH may actually represent lower respiratory tract infection, the detection of spores of facultative pathogenic and fungi such as *A. fumigatus* may reflect recent exposure to this ubiquitous mold and may not be an indicator of invasive diseases.

When compared with the other detection methods, FISH results offer potential clues for improving diagnostic tests for respiratory mold infections. First, FISH targeting rRNA was less sensitive than PCR targeting fungal DNA. This may be caused by a lack of fluorescence signal used to define the presence of conidia. *Aspergillus* conidia may represent metabolically inactive structures that are not identified by FISH due to a low rRNA copy number and therefore low fluorescence signal intensity. They may become identifiable by FISH after germination due to sensing of nutrients after inhalation, a process that occurs asynchronously in different conidia, leading first to swelling of conidia and later polarized growth as germ tubes and later mycelia in germinating conidia. A preincubation with nutrients or germination inducers may improve FISH positivity and may improve cultivation (23). Alternatively, PCR may frequently detect free DNA released by antifungals or host defense. Therefore, optimization of DNA extraction should include testing of conidia, free DNA, and hyphae. Indeed, DNA yield can be different for extraction kits used for conidia vs hyphae (24). Interestingly, protocols that do not have any fungal lysis step allow for fungal DNA detection in BALF, indicating that free DNA released through immune cells or antifungal therapy might be amplified from BALF (25). In conclusion, while recommendation for the detection of *Aspergillus* DNA from blood has been published by the FPCRI based on extensive studies, our data suggest that there is a need for further standardization of BAL testing with a potential role of FISH in defining the target of fungal DNA extraction to generate evidence-based recommendations (26).

Although FISH has the potential for sensitive and specific detection of fungi in BALF, it has also technical limitations. First, the use of short oligonucleotides offers more limited species resolution than PCR, potentially yielding outside hits, that is, failure to discriminate between closely related fungi. Second, if fluorescence is used for detection, autofluorescence of fungal material requires adequate controls to rule out false positives.

Coinfections with *Aspergillus* and Mucorales have been described in diverse patient populations, including hematologic malignancy, organ transplantation, diabetes, and severe viral pneumonia, by detection of these fungi predominantly in respiratory samples (BALF, tissue biopsy) and blood (27). Such infections may pose significant therapeutic challenges. When microbiologic tests on BALF are based on *Aspergillus* antigen and even culture, mucormycosis may not be detected, potentially leading to inadequate patient management. Previously, it had been demonstrated that Mucorales can be detected by qPCR in 9.5% of patients with probable invasive aspergillosis by qPCR (28, 29). Our finding that DNA load of Mucorales in BALF is not significantly higher than in serum confirms previous studies demonstrating the usefulness of qPCR from serum for the early diagnosis and assessment of treatment response in mucormycosis in patients with hematologic malignancy (30). However, as only five of eight patients with detection of Mucorales DNA in BALF and available serum were serum positive by Mucorales qPCR, BALF testing appears mandatory, in line with a sensitivity of 85% of serum Mucorales PCR published by Millon et al. (30).

Viral respiratory tract infections increase the risk of developing pulmonary aspergillosis, probably due to impairment of the epithelial barriers or inability to generate an effective immune response (3). While most research has been performed on CMV, influenza A virus, and recently SARS-CoV-2, other viruses have been reported mainly in transplant recipients. Among community-acquired respiratory viruses, rhinovirus has been previously shown to be the predominant virus cultivated from respiratory specimens in patients with hematologic malignancy, both with and without stem cell transplantation (31). A reliable detection enables patient isolation to prevent further transmission. However, in the absence of a viral pandemic during the study period, respiratory viruses and CMV were infrequently identified in our patients.

In conclusion, a combination of specific qPCRs from BALF is the most sensitive molecular test to identify *Aspergillus* and Mucorales in cancer patients with suspected mold pneumonia. The predominant detection of Mucorales DNA in the presence of *Aspergillus* DNA supports routine testing beyond *Aspergillus* in patients at high risk for fungal pneumonia. The use of FISH to visualize fungal structures in clinical samples may offer optimization of diagnostic tests.

## Data Availability

Data are available upon request from the authors.

## SUPPLEMENTAL MATERIAL

Supplemental methods and results jcm.01201-25-s0001.docx Additional methods on viral diagnostics, *in vitro* resistance, and molecular typing of *Aspergillus fumigatus*.

ASM does not own the copyrights to Supplemental Material that may be linked to, or accessed through, an article. The authors have granted ASM a non-exclusive, world-wide license to publish the Supplemental Material files. Please contact the corresponding author directly for reuse.
