# Supplementary material for: Molecular diagnostics in cancer patients with suspected respiratory mold infections
Source: J Clin Microbiol. 2025 Nov 26;63(12):e01201-25. doi: 10.1128/jcm.01201-25 (PMC12710353; doi:10.1128/jcm.01201-25)
Supplement: Supplemental methods and results — Additional methods on viral diagnostics, in vitro resistance, and molecular typing of Aspergillus fumigatus. [file jcm.01201-25-s0001.docx]

**Supplemental Methods**

*In vitro* resistance testing of cultivated molds was performed with microdilution according to CLSI M38 standard using adequate controls.

Nucleotides of respiratory viruses were amplified by specific qPCR assays targeting Rhino-, Adeno-, human Metapneumo-, Influenza A-, Parainfluenza-, Respiratory syncytial-, Boca-, endemic Corona viruses (OC43, HKU1, NL63, 229E), and CMV was amplified by qPCR targeting US17.

**Supplemental Results**

CSP typing documented seven different previously defined CSP types (t01: 3; t02: 2; t03: 4; t04A: 3; t05: 1; t09: 1; t13: 1). *In vitro* resistance testing identified azole resistance (itraconazole >16 mg/dl; voriconazole 2 mg/dl) in one of 15 (7%) isolates only.

In a subgroup of samples, CMV (n=166) and respiratory viruses (n=191) were detected by qPCR in BALF (**Table 2**). CMV was amplified in 26 of 166 samples (16%) and respiratory viruses were detected in 33 of 191 (17%) samples. Specific qPCR detected Adenovirus (n=2), Bocavirus (n=1), endemic Coronaviruses (n=6), Influenza virus (n=4), Metapneumovirus (n=3), Parainfluenza virus (n=6) and Rhinovirus (n=11).

CMV detection from BALF was not associated with proven/probable mold infection [9/57 (16%) vs 17/109 (16%) p=0,9] or positive *Aspergillus* PCR [4/34 (12%) vs. 22/132 (18%) p=0.4] or culture [2/12 (17%) vs. 24/154 (16%) p=0.9], or Mucorales PCR [0/8 (0%) vs. 26/158 (16%) p=0.2]. Respiratory viruses were detected in 33 of 191 samples (17%). Detection of respiratory viruses was not significantly associated with proven/probable aspergillosis [8/70 (11%) vs. 25/121 (21%) p=0.1] but less likely in positive *Aspergillus* PCR [3/42 (7%) vs. 30/149 (20%) p=0.05] and Mucorales qPCR [1/9 (11%) vs. 32/182 (18%) p=0.6].
